# County Differences in Mortality among Foreign-Born Compared to Native Swedes 1970–1999

**DOI:** 10.1155/2012/136581

**Published:** 2012-09-18

**Authors:** Björn Albin, Katarina Hjelm, Jan Ekberg, Sölve Elmståhl

**Affiliations:** ^1^School of Health and Caring Sciences, Linnaeus University, 351 95 Växjö, Sweden; ^2^Centre of Labour Market Policy Research (CAFO), School of Business and Economics, Linnaeus University, 351 95 Växjö, Sweden; ^3^Department of Health Sciences, Division of Geriatric Medicine, Lund University, 221 00 Lund, Sweden

## Abstract

*Background*. Regional variations in mortality and morbidity have been shown in Europe and USA. Longitudinal studies have found increased mortality, dissimilarities in mortality pattern, and differences in utilization of healthcare between foreign- and native-born Swedes. No study has been found comparing mortality among foreign-born and native-born Swedes in relation to catchment areas/counties. *Methods*. The aim was to describe and compare mortality among foreign-born persons and native Swedes during 1970–1999 in 24 counties in Sweden. Data from the Statistics Sweden and the National Board of Health and Welfare was used, and the database consisted of 723,948 persons, 361,974 foreign-born living in Sweden in 1970 and aged 16 years and above and 361,974 matched Swedish controls. *Results*. Latest county of residence independently explained higher mortality among foreign-born persons in all but four counties; OR varied from 1.01 to 1.29. Counties with a more rural structure showed the highest differences between foreign-born persons and native controls. Foreign-born persons had a lower mean age (1.0–4.3 years) at time of death. *Conclusion*. County of residence influences mortality; higher mortality is indicated among migrants than native Swedes in counties with a more rural structure. Further studies are needed to explore possible explanations.

## 1. Introduction

Earlier studies have shown that mortality and morbidity vary in different parts of a country both in European countries and the US [1–4]. To our knowledge no study has compared mortality among foreign-born and native-born Swedes in relation to catchment areas such as counties. Geographical variation in mortality is due to influencing factors, in childhood and in adulthood, such as social class, employment status and social and physical environment [5], and unequal utilization of healthcare. Districts with high or low mortality could be identified in the UK [1]. Another investigation showed an association between area of residence and stomach cancer or stroke [3]. In Italy higher mortality for men was found in the north of the country compared to the south but the reversed pattern was evident for women [2]. 

Previous longitudinal studies of mortality, morbidity, and heath care utilization among foreign- and native-born Swedes during 1970–1999 have found increased mortality, dissimilarities in mortality pattern, and differences in utilization of hospital care [6–8]. The results showed higher mortality and a 2.5 to 2.8 years lower mean age at time of death for foreign-born persons compared to the Swedish controls. The mortality pattern showed a significantly higher number of deaths among foreign-born from* neoplasm*, *diseases of the circulatory system*, *symptoms, signs, and ill-defined conditions,* and *injury and poisonin*g, according to the International Classification of Diseases, ranging from 1.0% to 5.3% higher prevalence [7]. A tendency towards less healthcare utilization among migrants, especially men, as regards to the diagnosis groups *symptoms, signs, and ill-defined conditions* and *injury and poisoning *has been found [8].

In Sweden, geographical differences in mortality and morbidity of the total population have been described [9, 10]. The average lifetime was found to be longer in the southern part of Sweden. Cancer was more frequent in the three largest cities, whereas accident as cause of death was more often found in rural areas. Differences in utilization of healthcare in different age groups among elderly persons have been studied [11]. The study found fewer days in hospital care and fewer visits to a general practitioner in the oldest age group, 80+, during the last year before death.

Swedish society has changed during the last century due to international migration and foreign-born people living in Sweden constituted 2004 11.5 percent of the total population [12]. The population of foreign-born in Sweden shows a great mixture of different nationalities but is dominated by labour migrants from the Nordic countries, especially Finland, and European countries such as the former Yugoslavia, Germany, and Poland [12]. 

The differences in health found in previous investigations, among foreign-born and native Swedes, could be a general pattern or vary in different parts of Sweden. Whether differences in healthcare services between county council areas are related to mortality among foreign born is unknown. In accordance with the Swedish Health and Medical Services Act, there should be no differences in healthcare services [13]. Earlier findings and the lack of studies in the area of geographical differences in mortality among foreign-born and native population warrant further studies.

The aim of this study was to describe and compare the county distribution of mortality among foreign- and Swedish-born persons during the years 1970–1999. The pattern will be discussed in relation to gender, year of birth, and age at death, testing the null hypothesis that no county differences in mortality exist between foreign-born and native Swedes. 

## 2. Material and Methods

Data from Statistics Sweden (SCB) and the National Board of Health and Welfare Centre for Epidemiology covering the period 1970–1999 was analysed. The study population consisted of all 361,974 foreign-born persons aged 16 years and upward who were registered as living in Sweden in 1970, together with 361,974 matched Swedish controls for each person. The control was matched and was similar in age (±3 year), sex, occupation, type of employer, and lived in the same county in 1970. Type of employment was divided into three groups (government, municipal, or other employer). Occupation was coded according to the Nordic Occupation Classification System (NYK), and county represented all the 24 county council areas in Sweden. This data relates to the situation on 1 November 1970 and was taken from the National Census of 1970, which was a total census and checked against the National Population Register (RTB), which included data up to 31 December 1999. Each person was given a code if they were deceased, still living in Sweden, had emigrated, or if no information was available. Information from the National Board of Health and Welfare Centre for Epidemiology on date of death and death diagnosis was added to the database. In total 906,564 people were included, 50 percent foreign-born persons.

A Swedish matched control could not be found for 20,518 of the foreign-born persons due to the matching criteria. Exclusion criteria were as follows: if no information was available or if a person had emigrated or migrated back (“remigrated”), thus in total 163,896 persons were excluded from the database. Persons were then also excluded if the information from the control subject was missing due to migration. In total 182,614 persons, 109,458 men and 73,156 women, were excluded from the database; see Table 1. 

The largest group of excluded foreign- born persons, 45,228 persons, consisted of persons born in Finland. In the original database 44,067 of them were coded as migrated (remigrated) and mortality among 37,904 (86%) of them could be analysed with data from the Population Register Centre in Finland. The analysis showed the same mortality risk among the excluded group as the Finnish-born persons who stayed in Sweden [6].

The database used for analysis finally consisted of 723,948 persons, 361,974 foreign-born and 361, 974 Swedish controls. Latest county of residence was used to distribute the study population in different geographical areas in Sweden. Mobility in Sweden between different counties showed a similar pattern. Among persons still living in 1990, 27.2% of foreign-born and 25.5% of Swedes had lived in the same county during 1970–1990 (*P* < 0.001) and 81.1% versus 85.52% might have moved during the study period but were living the same county in 1990 as in 1970.

### 2.1. Characteristics of County Council Areas in Sweden

The 24 counties in Sweden differ with regard to population and area. The population varies from the largest one (Stockholm) with 1,860,872 inhabitants to the smallest (Gotland) with 57,535 inhabitants (SCB, 2004). The four most densely populated counties are, as in 1970, Stockholm, Göteborg och Bohus, Malmöhus, and Östergötland. Gotland, Blekinge, Kronoberg, and Kalmar represent the less populated and could be considered as more rural counties according to the national classification [14]. The different counties also vary in terms of area, which tends to be larger in the north of Sweden, Norrbotten is the largest, covering an area of 98,249.5 km^2^. In contrast, the smallest county (Blekinge) has an area of 2,946.7 km^2^.

### 2.2. Statistical Analysis

Values from the total cohort sample are given as numbers, means, and percentages. Comparisons were made by tests of significance with Mann-Whitney *U* test and Chi-square test. A value of *P* < 0.05 was considered statistically significant [15]. Logistic regression analysis was performed and a model tested, adjusted for age and sex, with the two independent variables foreign-born and latest county of residence (categorical) and the dependent variable dead or alive. Uppsala County, with the lowest mortality, was used as reference.

Cox regression analysis was performed in two age groups: persons aged 60–69 years and 80 years or more. The analysis was adjusted for sex and age and tested the importance of being foreign born and latest county of residence (categorical). Uppsala was also in this analysis used as reference.

All analyses were performed using SPSS (Statistical Package for Social Sciences), version 11.5. 

### 2.3. Ethics

Approval of the study was granted from the Ethics Committee of Lund University, Sweden, after a reviewing process from all other university ethics committees in Sweden. 

## 3. Results

### 3.1. Characteristics of Counties and Differences in Mortality in relation to being a Foreign-Born Person

The study population was geographically distributed among the 24 different county council areas that made up the regional organization of Sweden in 1970. The major groups of foreign-born persons were found in the counties of Stockholm, Göteborg och Bohus, and Malmöhus; see Table 2. There were no significant differences between foreign- and Swedish-born persons in the different counties in relation to sex. In four counties (Stockholm, Göteborg & Bohus, Västmanland, and Norrbotten), foreign-born persons had a significantly lower mean age than Swedish-born persons. The mean age was significantly higher among foreign-born men than Swedish-born men in eight and significantly lower in three counties. Foreign-born women had a higher mean age in nine and a lower mean age in three compared to Swedish-born women (Table 3).

During the studied period 1970–1999, a total of 116,063 foreign-born and 104,865 Swedish control persons had died. There was significant higher mortality for foreign-born persons than native Swedes in all counties with one exception, Norrbotten. The highest percentage of deceased among foreign-born persons was found in Gotland (43%), Värmland (40.0%), and Jämtland (37.4%). The largest difference in deceased subjects, with higher mortality among foreign-born than Swedish-born persons, was found in the rural counties of Gotland (7.6%), Blekinge (7.1%), Kalmar (5.9%), and Kronoberg (5.7%).

### 3.2. Influence of Gender on Mortality

There was significant higher mortality in all counties for men and in 19 of the 24 counties for women (Tables 4(a) and 4(b)). The highest proportion of deceased foreign-born men was found in Gotland (49.7%), Värmland (45.3%), and Jämtland (41.3%), see Table 4(a). The largest difference in percentage between deceased foreign- and Swedish-born male persons was in Gotland (11.4%), Blekinge (9.0%), Jämtland (7.9%), and Jönköping (7.6%). Foreign-born women had the highest percentage of deceased in Gotland (39.3%), Värmland (36.0%), and Norrbotten (32.8%). The difference in percentage between foreign- and Swedish-born deceased female persons was largest in the rural counties of Kalmar (5.3%), Blekinge (5.2%), Kronoberg (4.8%), and Gotland (4.7%); see Table 4(b).

### 3.3. Influence of Age on Mortality

Mean age at time of death was significantly lower for foreign- than Swedish-born men in all counties, ranging from 1.0 to 4.4 years, except for Kalmar and Gotland (Table 5). For women significant differences in mean age at time of death were found in 19 counties. There was a larger difference in mean age at time of death between foreign- and Swedish-born men than between foreign- and Swedish-born women in all counties. Differences in survival time between foreign- and Swedish-born persons were studied in particular in two age groups of older persons, those aged 60–69 and 80+, using Cox regression analysis. In the age group 60–69 years, foreign-born persons had a significantly lower survival time in two counties, Norrbotten and Värmland. No difference was found in any of the other counties. In the age group 80+ no significant differences were found.

### 3.4. Multifactorial Influence on Mortality

Logistic regression analysis studying the influence of being foreign-born and latest county of residence on mortality showed that being foreign-born was, adjusted for age and sex, an independent influencing factor (*P* = 0.000, *B* = 0.242), as was county, except in four county council areas (Kronoberg, Kalmar, Kristianstad, Halland and Skaraborg) and the OR varied from 1.01 to 1.29; see Table 6. 

## 4. Discussion 

The major findings in this study were that latest county of residence was an independent factor influencing mortality and that a variation with significantly higher mortality was found in all but one Swedish county among foreign-born persons compared with native Swedes. The highest difference compared to native controls of deceased foreign-born was found in counties with a more rural structure. Several factors might explain the noted differences, such as the size of the foreign-born population, differences in the healthcare system's ability to deliver healthcare, social network, stress, and economic resources.

## 5. Strengths and Limitations

The study of regional differences in mortality among foreign-born and native Swedes 1970–1999 was based on data from Statistics Sweden and from the National Board of Health and Welfare Centre for Epidemiology. 

The data used to establish the database originated from the Population and Housing Census of 1970, which is considered to be a total census as it was compulsory by law to take part. The number of dropouts has not been estimated for the total census, only for some of the variables such as “occupation”, which Statistics Sweden estimates to be 3.5–4.5%. One can only speculate about whether participation in the census is related to health problems and whether dropout might differ between foreign-born and native healthy Swedes. It seems unlikely that the proportion of healthy foreign-born should be higher than native Swedes and thereby introduce selection bias. Another reason why migrants do not participate in the census could be language problems.

A Swedish matched control could not be found for 20,518 of the foreign-born persons due to the matching criteria. The geographical distribution of the excluded persons was not different from the distribution in the database used.

The excluded group varies in proportion with regard to country/region of birth. Excluded persons from Finland constitute the largest proportion (30.8%). A followup of Finns who had remigrated was performed and showed no differences in mortality from the group of Finns included in the study [6]. Persons born in Finland constitute the largest group of migrants living in Sweden and were also the largest migrant group in the database used. There are no reasons to believe that “remigrants” born in other countries differ from the Finnish group. 

Selection bias has to be considered. The excluded persons had a lower mean age than the persons in the database, with the exception of persons born in the former Yugoslavia, but their Swedish control person of the same age was also excluded. Only 8.1% of the excluded persons had reached the age of 60–69 and 2.6% the age of 80+. There is no reason to assume that the excluded persons influenced the survival analysis using these two age groups, due to the low proportion of excluded persons above 60 years of age.

Latest county of residence has been used as a variable. Migration within Sweden might have occurred and labour migration by healthier persons to urban areas could explain the higher mortality in rural counties. However, the pattern of internal migration within Sweden showed similarities between foreign-born and native Swedes. Among foreign-born persons still living in Sweden 1990, 53.9% had changed county of residence during 1979–1999 but moved back to the same county 1990 as the one they lived in 1970, corresponding proportion for native Swedes was 60%.

## 6. Results

The rural counties had in common that the proportion of foreign-born persons in their populations was low compared to the more urban counties. For some ethnic groups higher density in an area of persons from the same ethnic group has been shown to have a positive effect on self-rated health, limiting long-standing illness [16], and reduce odds of infants mortality [17]. Belonging to a small migrant group may be a risk factor; earlier studies have shown higher hospital rates for mental health problems in areas where migrants constitute a small proportion of the community [18]. The result has been interpreted as showing that if you belong to a larger migrant group it is likely that the stress connected with being a migrant could be reduced due to stronger social network [18]. Stress is also known to increase the susceptibility to other diseases such as diabetes and hypertension [19, 20]. Furthermore, the ability of the healthcare systems, both hospitals and outpatient facilities, to handle and to investigate symptoms and signs among migrants is most likely to be poorer in counties with few migrants. It could be hypothesized that delay or misunderstanding in diagnosis and treatment and healthcare staff with insufficient “language skills” and understanding of cultural differences could influence mortality. In a previous study it was shown that symptoms, signs, and ill-defined condition were more common as cause of death among foreign-born compared to native Swedish persons [7].

Another explanatory factor could be differences in social networks in different geographical areas [21]. Poor social network and low social support are more common among foreign-born persons [22] and have been shown to influence and increase mortality [23].

Generally no significantly higher mortality among migrants was found in Norrbotten, but mortality was higher for men when men and women were analysed separately. 

This county has close cultural contacts with Finland [24] and many inhabitants are bilingual. A short cultural distance between migrants and native population has been described as important for health [25].

The differences in mortality in all Swedish counties, with one exception, among foreign-born persons compared with native Swedes could indicate differences in economic resources and needs in the population. One measure of economic resources in the county was the local tax rate. In the middle of the studied period (1985), local tax per unit differed from 28.46% (Kristianstad) to 32.00% in Blekinge of taxable income [26], although the taxation differences were also equalized by a national taxation transfer system [27]. Whether differences in economic resources between counties might influence the noted mortality differences in relation to foreign-born persons requires further analysis.

More healthcare needs are connected with an elderly population [10] and in 1985 the share of the population aged 80 years or more in the counties varied from 2.7% to 4.2% [27]. The lowest percentage for this age group was found in Norrbotten. The counties of Gotland and Blekinge, with the largest proportion of deceased foreign-born persons, were also characterized in 1985 by a low percentage of foreign-born persons (2.3% and 5.1%) and average or higher than average percentage of the population 80 aged years or more (3.8% and 3.9%).

There are a strong connection between increased mortality and increased age [9, 10, 12]. A higher mean age in the foreign-born population could also explain the larger proportion of deceased foreign-born women in two counties (Kalmar and Gotland) but was not found in the counties of Kronoberg and Blekinge. Furthermore, the variation in mortality between counties with higher mortality among foreign-born remained after adjustment for age and gender.

Other explanatory factors could be work environment and type of employment. Foreign-born men have to a higher degree been employed in private manufacturing industry, which is often found in rural counties like Kronoberg, Kalmar, and Blekinge [28]. Foreign-born women in Sweden could to a higher extent be exposed to shift work and physical and stressful work [29, 30].

In most counties and especially for men, the mean age at time of death is lower among foreign-born persons than native Swedes. The mean age at time of death of foreign- born men in the two counties Norrbotten and Värmland with highest OR was 2.4 to 3.7 years lower than native Swedes and correspondingly 1.3 to 1.8 years lower for women. Cox regression analysis also confirms a lower survival time for foreign-born persons in the counties of Norrbotten and Värmland for the age group 60–69. 

## 7. Conclusion

In conclusion, county of residence influences mortality, and the study indicates a tendency to higher mortality among foreign-born persons than native Swedes in counties with a more rural structure. Further studies are needed to explore possible explanations and to establish the county-specific characteristics to explain variations between county council areas.

## Figures and Tables

**Table 1:** Excluded and analysed persons related to country of birth, sex, and age.

	Excluded male persons			Analysed male persons		Excluded female persons			Analysed female persons	
	*n*	Mean age 1970	Proportion excluded %	*n*	Mean age 1970	*n*	Mean age 1970	Proportion excluded %	*n*	Mean age 1970
Swedish	54 729	31.5	24.5	168 702	39.4	36 578	32.2	15.9	193 272	42.0
Denmark	3 285	34.8	17.4	15 627	43.0	2 054	34.4	13.0	13 795	44.1
Finland	27 193	29.3	30.8	60 959	34.8	18 035	29.9	17.9	82 544	37.4
Norway/Iceland	3 654	32.9	21.6	13 236	45.7	2 875	33.4	11.4	22 413	48.0
Yugoslavia	3 518	32.6	23.3	11 598	31.8	2 519	33.1	23.2	8 362	31.1
Poland	462	35.4	9.8	4 253	44.6	354	35.2	6.7	4 925	44.6
Germany	2 613	34.6	15.5	14 291	41.6	2 123	36.4	9.7	19 675	45.0
Other European	10 442	34.2	23.2	34 510	42.0	6 577	34.9	18.9	28 229	46.0
Non-European	3 496	32.4	19.9	14 035	45.0	1 966	33.9	13.1	13 081	51.7
Stateless/unknown	66	30.6	25.5	193	50.2	75	30.7	23.2	248	55.8

Total	109 458		100	337 404		73 156			386 544	

**Table 2:** Population in relation to sex and latest County Council of residence.

County council	Proportion of foreign-born of total population 1970	Men foreign-born		Swedish-born			Women foreign-born		Swedish-born		
	%	*n*	%	*n*	%	*P*-value	*n*	%	*n*	%	*P*-value
Stockholm	10.5	50 683	45.5	45 955	45.1	0.218	60 677	54.5	55 986	54.9	0.278
Uppsala	6.1	4 016	46.5	4 178	46.3	0.512	4 614	53.5	4 849	53.7	0.278
Södermanland	9.5	7 100	49.1	7 271	49.6	0.658	7 350	50.9	7 394	50.4	0.663
Östergötland	5.0	5 444	47.8	6 048	47.9	0.913	5 942	52.2	6 570	52.1	0.918
Jönköping	5.1	4 180	46.9	4 593	47.1	0.868	4 728	53.1	5 153	52.9	0.878
Kronoberg	5.0	2 117	48.6	2 626	49.8	0.504	2 238	51.4	2 649	50.2	0.513
Kalmar	3.4	2 352	48.5	2 915	47.9	0.746	2 502	51.5	3 167	52.1	0.758
Gotland	2.5	441	38.1	561	38.8	0.811	717	61.9	886	61.2	0.861
Blekinge	5.1	1 957	49.0	2 329	49.7	0.701	2 035	51.0	2 354	50.3	0.706
Kristianstad	3.8	3 543	46.3	3 832	46.1	0.876	4 112	53.7	4 484	53.9	0.888
Malmöhus	7.3	17 990	48.8	17 002	48.6	0.778	18 893	51.2	17 984	51.4	0.785
Halland	4.7	3 583	46.8	3 887	47.3	0.685	4 079	53.2	4 331	52.7	0.708
Göteborg och Bohus	8.4	19 041	50.5	18 097	50.0	0.439	18 664	49.5	18 093	50.0	0.436
Älvsborg	7.8	8 761	44.7	9 121	44.9	0.868	10 827	55.3	11 180	55.1	0.757
Skaraborg	3.9	2 966	46.2	3 339	46.3	0.945	3 452	53.8	3 871	53.7	0.950
Värmland	4.6	4 365	43.5	4 782	44.5	0.338	5 681	56.5	5 962	55.5	0.415
Örebro	6.2	5 148	48.7	5 568	48.8	0.923	5 418	51.3	5 834	51.2	0.925
Västmanland	11.6	8 931	50.1	8 653	50.6	0.602	8 900	49.9	8 457	49.4	0.599
Dalarna	4.8	4 805	48.4	5 535	48.3	0.978	5 126	51.6	5 913	51.7	0.977
Gävleborg	3.7	3 468	49.0	4 027	48.9	0.976	3 611	51.0	4 200	51.1	0.977
Västernorrland	2.5	2 365	44.1	2 675	44.9	0.601	2 996	55.9	3 282	55.1	0.651
Jämtland	2.3	973	39.2	1 344	42.7	0.088	1 511	60.8	1 807	57.3	0.180
Västerbotten	1.9	1 323	35.7	1 622	37.9	0.163	2 383	64.3	2 655	62.1	0.329
Norrbotten	4.5	3 150	31.6	2 742	30.6	0.292	6 816	68.4	6 211	69.4	0.532

Total		168 702		168 702			193 272		193 272		

**Table 3:** Population in relation to age (1970) and latest county council of residence.

County council	Foreign-born men		Swedish-born men			Foreign-born women		Swedish-born women		
	Age (mean)	95% CI	Age (mean)	95% CI	*P* value	Age (mean)	95% CI	Age (mean)	95% CI	*P* value
Stockholm	39.9	39.8, 40.0	40.6	40.4, 40.7	0.000	43.0	42.9, 43.2	43.7	43.6, 43.8	0.000
Uppsala	36.6	36.2, 37.1	36.7	36.2, 37.1	0.884	39.2	38.7, 39.6	39.0	38.6, 39.4	0.524
Södermanland	38.5	38.2, 38.8	38.6	38.3, 38.9	0.670	40.0	39.7, 40.4	40.2	39.9, 40.6	0.487
Östergötland	38.8	38.4, 39.1	38.1	37.7, 38.4	0.008	40.9	40.5, 41.3	40.4	40.0, 40.7	0.037
Jönköping	38.3	37.8, 38.7	37.6	37.2, 38.1	0.026	40.5	40.0, 40.9	39.9	39.5, 40.3	0.029
Kronoberg	39.0	38.4, 39.7	38.3	37.7, 38.8	0.088	40.9	40.3, 41.6	40.1	39.5, 40.7	0.055
Kalmar	40.6	40.0, 41.2	39.0	38.4, 39.5	0.000	42.9	42.3, 43.5	41.0	40.4, 41.5	0.000
Gotland	45.7	44.1, 47.3	42.6	41.2, 44.0	0.002	47.7	46.5, 48.9	44.6	43.5, 45.8	0.000
Blekinge	39.4	38.8, 40.1	38.1	37.5, 38.7	0.001	42.1	41.3, 42.8	41.1	40.2, 41.8	0.068
Kristianstad	40.6	40.1, 41.0	39.7	39.3, 40.2	0.009	42.6	42.1, 43.1	41.8	41.4, 42.3	0.022
Malmöhus	39.7	39.5, 40.0	39.8	39.6, 40.0	0.725	42.5	42.3, 42.8	42.7	42.5, 43.0	0.377
Halland	38.6	38.2, 39.1	38.2	37.7, 38.6	0.093	40.8	40.3, 41.3	40.1	39.7, 40.6	0.037
Göteborg och Bohus	39.1	38.9, 39.3	39.5	39.3, 39.7	0.002	42.3	42.1, 42.6	42.6	42.4, 42.9	0.080
Älvsborg	39.0	38.7, 39.2	38.7	38.4, 39.0	0.071	40.8	40.5, 41.1	40.6	40.3, 40.9	0.172
Skaraborg	37.6	37.1, 38.1	37.0	36.5, 37.5	0.074	40.0	39.5, 40.5	39.4	38.9, 39.9	0.061
Värmland	44.1	43.6, 44.6	43.0	42.6, 43.5	0.001	45.4	45.0, 45.8	44.8	44.3, 45.2	0.034
Örebro	38.9	38.5, 39.3	38.6	38.2, 38.9	0.195	40.9	40.5, 41.3	40.5	40.1, 40.9	0.122
Västmanland	37.7	37.4, 38.0	38.0	37.6, 38.3	0.138	39.0	38.7, 39.3	39.5	39.2, 39.8	0.009
Dalarna	39.3	39.0, 39.7	39.0	38.7, 39.4	0.253	41.0	40.6, 41.4	40.5	40.2, 40.9	0.072
Gävleborg	38.0	37.6, 38.5	37.6	37.2, 38.0	0.230	40.6	40.1, 41.1	39.8	39.4, 40.3	0.038
Västernorrland	38.6	38.1, 39.2	38.5	38.0, 39.0	0.880	42.0	41.4, 42.5	41.5	40.9, 42.0	0.222
Jämtland	42.8	41.9, 43.8	40.4	39.6, 41.2	0.000	44.1	43.3, 44.8	42.5	41.8, 43.2	0.002
Västerbotten	37.8	37.0, 38.6	37.0	36.4, 37.7	0.389	41.4	40.8, 42.0	41.2	40.6, 41.7	0.625
Norrbotten	38.8	38.3, 39.3	39.7	39.1, 40.3	0.022	43.5	43.1, 43.9	44.7	44.3, 45.1	0.000

Total										

**Table:** (a)

County council	Foreign-born men		Swedish-born men			
	*n*	%	n	%	P-value	% difference
Gotland	219	49.7	215	38.3	0.000	11.4
Blekinge	697	35.6	620	26.6	0.000	9.0
Jämtland	402	41.3	449	33.4	0.000	7.9
Jönköping	1 366	32.7	1 155	25.1	0.000	7.6
Västernorrland	809	34.2	723	27.0	0.000	7.2
Kronoberg	700	33.1	686	26.1	0.000	7.0
Älvsborg	3 060	34.9	2 542	27.9	0.000	7.0
Örebro	1 776	34.5	1 553	27.9	0.000	6.6
Östergötland	1 821	33.4	1 632	27.0	0.000	6.4
Kalmar	830	35.3	841	28.9	0.000	6.4
Stockholm	18 214	39.9	15 613	34.0	0.000	5.9
Värmland	1 976	45.3	1 899	39.7	0.000	5.6
Södermanland	2 482	35.0	2 142	29.5	0.000	5.5
Kristianstad	1 214	34.3	1 105	28.8	0.000	5.5
Skaraborg	891	30.0	829	24.8	0.000	5.2
Dalarna	1 668	34.7	1 634	29.5	0.000	5.2
Halland	1 077	30.1	981	25.2	0.000	4.9
Gävleborg	1 149	33.1	1 134	28.2	0.000	4.9
Göteborg och Bohus	6 816	35.8	5 610	31.0	0.000	4.8
Västerbotten	392	29.6	422	26.0	0.029	3.6
Malmöhus	6 311	35.1	5 354	31.5	0.000	3.6
Västmanland	2 737	30.6	2 343	27.1	0.000	3.5
Uppsala	1 117	27.8	1 026	24.6	0.001	3.2
Norrbotten	1096	34.8	883	32.2	0.036	2.6

Total	58 820		51 391	

**Table:** (b)

County council	Foreign-born women		Swedish-born women			
	n	%	n	%	P-value	% difference
Kalmar	770	30.8	807	25.5	0.000	5.3
Blekinge	640	31.4	617	26.2	0.000	5.2
Kronoberg	617	27.6	604	22.8	0.000	4.8
Gotland	282	39.3	301	34.0	0.027	4.7
Kristianstad	1 239	30.1	1 155	25.8	0.000	4.3
Jönköping	1 220	25.8	1 152	22.4	0.000	3.4
Älvsborg	3 009	27.8	2 768	24.8	0.000	3.0
Gävleborg	982	27.2	1 015	24.2	0.002	3.0
Östergötland	1 647	27.7	1 632	24.8	0.000	2.9
Malmöhus	5 791	30.7	5 014	27.9	0.000	2.8
Halland	1 028	25.2	977	22.6	0.004	2.6
Västerbotten	669	28.1	681	25.6	0.052	2.5
Örebro	1 483	27.4	1 466	25.1	0.007	2.3
Dalarna	1 397	27.3	1 478	25.0	0.007	2.3
Göteborg och Bohus	5 723	30.7	5 200	28.7	0.000	2.0
Uppsala	1 099	23.8	1 057	21.8	0.019	2.0
Västernorrland	905	30.2	925	28.2	0.078	2.0
Värmland	2 046	36.0	2 040	34.2	0.042	1.8
Södermanland	1 954	26.6	1 846	25.0	0.025	1.6
Skaraborg	843	24.4	887	22.9	0.130	1.5
Norrbotten	2 235	32.8	2 090	33.6	0.298	0.8
Stockholm	19 140	31.5	17 289	30.9	0.015	0.6
Västmanland	2 065	23.2	1 927	22.8	0.515	0.4
Jämtland	459	30.4	546	30.2	0.920	0.2

Total	57 243		53 474			

**Table 5:** Deceased in relation to age at time of death and latest county council of residence.

County council	Foreign-born men		Swedish-born men			Foreign-born women		Swedish-born women		
	Age (mean)	95% CI	Age (mean)	95% CI	*P*-value	Age (mean)	95% CI	Age (mean)	95% CI	*P*-value
Stockholm	67.5	67.3, 67.7	70.6	70.4, 70.8	0.000	75.0	74.8, 75.2	76.9	76.7, 77.1	0.000
Uppsala	66.0	65.1, 66.8	69.1	68.2, 70.0	0.000	73.7	72.9, 74.5	75.2	74.4, 76.0	0.004
Södermanland	65.7	65.2, 66.3	70.1	69.5, 70.6	0.000	72.6	72.0, 73.2	74.8	74.2, 75.4	0.000
Östergötland	66.2	65.5, 66.9	70.5	69.9, 71.1	0.000	73.5	72.8, 74.2	75.1	74.4, 75.7	0.002
Jönköping	66.3	65.6, 67.1	70.2	69.5, 71.0	0.000	72.9	72.1, 73.7	74.2	73.4, 75.0	0.013
Kronoberg	68.5	67.4, 69.5	71.5	70.6, 72.5	0.000	73.3	72.1, 74.4	75.5	74.5, 76.5	0.008
Kalmar	68.7	67.7, 69.7	70.4	69.6, 71.3	0.060	75.5	74.6, 76.4	75.8	75.0, 76.7	0.626
Gotland	72.4	70.7, 74.1	73.6	71.9, 75.2	0.310	78.0	76.6, 79.3	77.9	76.6, 79.3	0.979
Blekinge	67.9	66.9, 68.9	71.3	70.3, 72.3	0.000	74.4	73.3, 75.5	76.6	75.7, 77.6	0.005
Kristianstad	69.7	68.9, 70.4	72.2	71.5, 72.9	0.000	74.9	74.2, 75.7	76.8	76.1, 77.5	0.001
Malmöhus	67.9	67.5, 68.2	70.6	70.3, 70.9	0.000	74.4	74.1, 74.8	76.7	76.4, 77.1	0.000
Halland	67.8	67.1, 68.6	70.7	69.9, 71.6	0.000	74.4	73.5, 75.2	76.3	75.5, 77.1	0.001
Göteborg och Bohus	65.9	65.5, 66.2	69.7	69.3, 70.0	0.000	73.9	73.6, 74.3	76.0	75.7, 76.3	0.000
Älvsborg	67.1	66.6, 67.7	71.3	70.8, 71.8	0.000	73.9	73.5, 74.4	76.1	75.6, 76.6	0.000
Skaraborg	67.0	66.0, 67.9	70.5	69.7, 71.4	0.000	73.4	72.4, 74.3	74.6	73.8, 75.4	0.105
Värmland	70.3	69.8, 70.9	72.7	72.2, 73.2	0.000	75.5	75.0, 76.1	76.8	76.3, 77.3	0.001
Örebro	66.6	65.9, 67.2	70.2	69.5, 70.8	0.000	72.3	71.7, 73.0	74.0	73.4, 74.7	0.000
Västmanland	65.7	65.2, 66.2	69.5	69.0, 70.0	0.000	71.8	71.2, 72.3	73.0	72.4, 73.6	0.002
Dalarna	66.6	65.9, 67.2	70.8	70.2, 71.4	0.000	72.5	71.8, 73.2	74.2	73.5, 74.8	0.001
Gävleborg	64.8	64.0, 65.6	68.6	67.9, 69.3	0.000	72.6	71.7, 73.4	74.5	73.7, 75.2	0.005
Västernorrland	66.4	65.5, 67.3	70.4	69.5, 71.3	0.000	73.7	72.8, 74.5	74.2	73.4, 75.1	0.340
Jämtland	70.3	69.0, 71.6	71.3	70.1, 72.4	0.000	75.3	74.2, 76.5	74.8	73.8, 75.8	0.558
Västerbotten	67.7	66.3, 69.1	70.0	68.8, 71.2	0.003	72.4	71.4, 73.4	74.4	73.5, 75.3	0.008
Norrbotten	67.1	66.3, 67.9	70.8	70.0, 71.6	0.000	74.7	74.2, 75.2	76.0	75.5, 76.5	0.000

**Table 6:** Multifactorial influence of being foreign-born on mortality adjusted for age and gender with latest county council of residence as independent categorical variable using Uppsala county council as reference.

County council	*B*-value	SE	*P*-value	OR	95% CI for Exp. *B*
Stockholm	0.180	0.023	0.000	1.197	1.144, 1.253
Södermanland	0.197	0.028	0.000	1.218	1.154, 1.286
Östergötland	0.116	0.029	0.000	1.123	1.061, 1.189
Jönköping	0.063	0.031	0.041	1.065	1.003, 1.131
Kronoberg	0.013	0.037	0.736	1.013	0.941, 1.089
Kalmar	0.062	0.035	0.079	1.064	0.993, 1.140
Gotland	0.194	0.059	0.001	1.214	1.081, 1.362
Blekinge	0.113	0.038	0.003	1.119	1.038, 1.206
Kristianstad	0.022	0.032	0.482	1.023	0.961, 1.089
Malmöhus	0.157	0.025	0.000	1.170	1.115, 1.228
Halland	−0.053	0.032	0.099	0.948	0.890, 1.010
Göteborg och Bohus	0.210	0.025	0.000	1.233	1.175, 1.294
Älvsborg	0.106	0.027	0.000	1.112	1.055, 1.172
Skaraborg	0.047	0.034	0.159	1.049	0.982, 1.120
Värmland	0.255	0.029	0.000	1.291	1.218, 1.367
Örebro	0.158	0.029	0.000	1.171	1.106, 1.240
Västmanland	0.087	0.027	0.001	1.091	1.035, 1.151
Dalarna	0.157	0.029	0.000	1.170	1.105, 1.239
Gävleborg	0.215	0.032	0.000	1.240	1.165, 1.319
Västernorrland	0.186	0.034	0.000	1.204	1.125, 1.288
Jämtland	0.194	0.043	0.000	1.214	1.116, 1.321
Västerbotten	0.126	0.039	0.001	1.134	1.051, 1.224
Norrbotten	0.230	0.030	0.000	1.259	1.187, 1.336
